# Interferon-inducible SAMHD1 restricts viral replication through downregulation of lipid synthesis

**DOI:** 10.3389/fimmu.2022.1007718

**Published:** 2022-11-30

**Authors:** Ni An, Qinghua Ge, Huihan Shao, Quanjie Li, Fei Guo, Chen Liang, Xiaoyu Li, Dongrong Yi, Long Yang, Shan Cen

**Affiliations:** ^1^ Institute of Medicinal Biotechnology, Chinese Academy of Medical Science, Beijing, China; ^2^ Institute of Pathogen Biology, Chinese Academy of Medical Science, Beijing, China; ^3^ Lady Davis Institute for Medical Research and McGill AIDS Centre, Jewish General Hospital, Montreal, QC, Canada; ^4^ Research Center for Infectious Diseases, Tianjin University of Traditional Chinese Medicine, Tianjin, China

**Keywords:** SAMHD1, lipid droplets (LDs), SREBP1, HCV, interferon

## Abstract

**Background:**

Type I interferon (IFN) inhibits virus infection through multiple processes. Recent evidence indicates that IFN carries out its antiviral activity through readjusting of the cellular metabolism. The sterile alpha motif and histidine-aspartate domain containing protein 1 (SAMHD1), as an interferon-stimulated gene (ISG), has been reported to inhibit a number of retroviruses and DNA viruses, by depleting dNTPs indispensable for viral DNA replication. Here we report a new antiviral activity of SAMHD1 against RNA viruses including HCV and some other flaviviruses infection.

**Methods:**

Multiple cellular and molecular biological technologies have been used to detect virus infection, replication and variation of intracellular proteins, including western blotting, qRT-PCR, Gene silencing, immunofluorescence, etc. Besides, microarray gene chip technology was applied to analyze the effects of SAMHD1 overexpression on total expressed genes.

**Results:**

Our data show that SAMHD1 down-regulates the expression of genes related to lipid bio-metabolic pathway, accompanied with impaired lipid droplets (LDs) formation, two events important for flaviviruses infection. Mechanic study reveals that SAMHD1 mainly targets on HCV RNA replication, resulting in a broad inhibitory effect on the infectivity of flaviviruses. The C-terminal domain of SAMHD1 is showed to determine its antiviral function, which is regulated by the phosphorylation of T592. Restored lipid level by overexpression of SREBP1 or supplement with LDs counteracts with the antiviral activity of SAMHD1, providing evidence supporting the role of SAMHD1-mediated down-regulation of lipid synthesis in its function to inhibit viral infection.

**Conclusion:**

SAMHD1 plays an important role in IFN-mediated blockade of flaviviruses infection through targeting lipid bio-metabolic pathway.

## Introduction

Viral replication is a high energy-consuming process that totally counts on host metabolism. Therefore, viruses have evolved to hijack and regulate synthesis of proteins, nucleic acids, and lipids to serve their infection and replication. To combat virus infection, the host has developed a highly complicated immunity system to monitor and maintain a normal metabolic process. Interferon (IFN) signaling, as an important member of innate immunity, enhances and induces a good deal of interferon-stimulated genes (ISGs) to restrain viral infection. Besides directly targeting viral component, they also join in the regulation of cellular metabolism, both of which produce inhibitive effects on viral infection. Recent studies have illuminated the specific roles of ISGs in adjusting cellular metabolism to contain viral infection. Several of these ISGs, the sterile alpha motif and histidine-aspartate domain containing protein 1 (SAMHD1), spermidine/spermine acetyltransferase 1 (SAT-1), cholesterol-25-hydroxylase (CH25H), and indoleamine-2,3-dioxygenase (IDO1), exert antiviral activity against multiple viruses by manipulating different metabolic pathways (1). For instance, CH25H is able to transfer cholesterol to the oxysterol 25-hydroxycholesterol that represents a well-defined regulator of sterol biosynthesis, thus exerting its antiviral activity (2). Upregulation of SAT-1 by IFNs results in downregulation of polyamine, thereby suppressing replication of polyamine-dependent viruses (3). Deprivation of L-tryptophan by IFN-inducible IDO1 results in a strong blockade of viral protein synthesis (4).

As a homologous gene of mouse Mg11, SAMHD1 gene was originally discovered in human dendritic cells (5). Subsequently, more studies indicated that SAMHD1 could be upregulated by different types of IFNs in various cells, especially in resting CD4+ T cells and myeloid cells (6). A recent study reveals that SAMHD1 possesses dNTP hydrolase activity and catalyzes the transformation of deoxynucleoside triphosphates to inorganic triphosphate and deoxynucleoside (7). The structure of SAMHD1 protein mainly comprises a sterile alpha motif (SAM), a histidine aspartic acid-containing domain (HD), and a C-terminus domain (8). The HD domain of SAMHD1 is the most important part for maintaining the oligomeric state of dNTPase, enhancing nucleic acid interaction and exerting antiviral activity (9). Depletion of dNTPs required for viral DNA synthesis was regarded as the main antiviral mechanism. Thereby, a wide range of retroviruses and DNA viruses, including HIV, T cell leukemia virus type 1, HBV, HSV-1, and vaccinia virus, were sensitive to the suppression of SAMHD1 (10–13). Although some experimental results prove that the anti-HIV-1 activity of SAMHD1 is closely related to intracellular dNTP levels, exogenous addition of dNTP does not completely relieve the inhibitory effect of SAMHD1 and restore the replication of HIV-1, indicating that SAMHD1 is likely to have a dNTPase-independent antiviral mechanism. Consistent with this notion, the phosphorylation of SAMHD1 at Threonine 592 located in the C-terminus peptide significantly affects the antiviral capacity, but not the dNTPase domain of SAMHD1 (14). Therefore, the mechanism underlying the antiviral activity of SAMHD1 awaits further investigation.

The life cycle of the flavivirus is closely related with the host lipids that are involved in viral entry, RNA replication, and assembly (15). Because they lack their own machinery to execute lipid synthesis, flaviviruses have to hijack host lipids to complete their intracellular replication. They also enhance cholesterol and fatty acid (FA) synthesis to generate viral membranes and produce ATP, suggesting that such viruses could manipulate lipid synthesis (16, 17). LDs are ER-related organelles associated with diverse cellular processes including lipid trafficking, immunity, cellular signaling, and virus replication (18–20). Lysosomes degrade LDs to release stored lipids for energy supply, which is effectively and efficiently hijacked by flaviviruses to support their own replication (21). Therefore, it is conceivable that type I IFN signaling may regulate FA and cholesterol synthesis and thereby prevent the infection of flavivirus and other viruses that depend on lipid synthesis. In agreement with this, 25-hydroxycholesterol (25-HC), a secreted IFN-induced protein, exhibits its wide-ranging antiviral functions through inhibiting sterol regulatory element binding protein (SREBP1) activation (22). SREBP1 is involved in enhancing cholesterol production by increasing the intake of LDL and synthesis of cholesterol (23).

In this work, we provide evidence showing that SAMHD1 plays a new role in negatively regulating both SREBP1 expression and LD formation, impairs HCV RNA replication, and inhibits the infectivity of HCV and other flavivirus. This work suggests that SAMHD1 may act as an innate immune effector to restrict flaviviridae family and other lipid-dependent viruses.

## Materials and methods

### Plasmid construction

Full-length SAMHD1 cDNA and the cDNA fragment encoding SAMHD1 truncations (base pairs 1–582, 45–626, and 112–626 of SAMHD1 cDNA) with Myc tag sequences were cloned into the pcDNA4.0 vector (Invitrogen) using the *Kpn*I and *Eco*RI restriction sites. SAMHD1 point mutations (T592A and T592D) were generated by using a site-directed mutagenesis kit (SBS). The plasmid of infectious HCV DNA clone (JFH1) was kindly offered by Takaji Wakita. The HCV 5’ untranslated region (UTR) and 3’UTR sequences were cloned into the 5’-terminus and 3’-terminus of the Renilla luciferase reporter gene, respectively, to construct a reporter gene translation system mediated by HCV IRES. The primers were designed as follows: for 5’UTR, 5’-CCCAAGCTTACCTGCCCCTAATAGGGGCG-3’/5’-CGGGATCCGTTGGTGTTTCTTTTGGT-3’; for Rluc, 5’-CGGGATCCATGACCAGCAAGGTGTACGA-3’/5’-CCGCTCGAGTTACTGCTCGTTCTTCAGCA-3’; for 3’UTR, 5’-CCGCTCGAGAGCGGCACACACTAGGTACA-3’/5’-GGGCCCACATGATCTGCAGAGAGACC-3’; and for T7-Rluc, 5’-TAATACGACTCACTATAGGACCTGCCCCTAATAGGGGCG-3’/5’-CCGCTCGAGTTACTGCTCGTTCTTCAGCA-3’.

### Cell culture and transfection

All cells including Huh7.5.1 cells (kept in our lab), Huh7 cells (kept in our lab), Vero cells (CCL-81; ATCC), Huh7 cell line containing the JFH1-derived subgenomic replicon (JFH1; HCV subtype 2a) (kept in our lab), and HEK293T cells (CRL-11268; ATCC) were cultured in Dulbecco’s modified Eagle’s medium (DMEM) (Gibco) with 10% fetal bovine serum (FBS) at 37°C with 5% CO_2_. Plasmids and mRNA were respectively transfected into cells with Lipofectamine 2000 (Invitrogen) and Vigofect (Vigorous) in line with the manufacturer’s instructions.

### Production of JFH1 HCVcc

JFH1 (HCV subtype 2a) mRNA was produced by using an *in vitro* RNA transcription kit (Ambion) according to the manufacturer’s instructions and then transfected into Huh7.5.1 cells with Lipofectamine RNAi Max (Invitrogen). At 72 hpt, culture supernatants were collected for cell debris removement and further concentration and then stored at −80°C. The 50% tissue culture infective dose (TCID_50_) of the HCV stock was determined by gradient dilution assay and immunofluorescence staining.

### Virus infections

A total of 5 × 10^5^ per well of Huh7.5.1 cells (or SAMHD1-KD Huh7.5.1 cells) were seeded into six-well plates for 24 h before transfection with pSAMHD1. At 48 hpt, JFH1 HCVcc was used to infect cells overexpressing SAMHD1 at an MOI = 0.2. After 72 h of incubation, cells were harvested for virus (or host) proteins or RNA analysis and supernatants were used for progeny virus analysis. Vero cells were used to infect cells with Japanese encephalitis virus (JEV) (SA14-14-2) or dengue virus 2 (DENV2) (Tr1751). JEV RNA and DENV2 titer were respectively measured by qRT-PCR and plaque assay to determine their own infection level.

### Western blotting

Cells were lysed in cell lysis solution (Pierce) on ice for 1 h and centrifuged at 12,000 rpm for 5 min to remove cell debris. The supernatants were subjected to SDS-PAGE. Proteins were then transferred to a 0.45-μm polyvinylidene difluoride (PVDF) membrane and labeled with specific antibodies. The antibodies used in this subject were as follows: anti-MYC (9E10, 1:1,000), anti-HA (H9658, 1:1,000), anti-FLAG (F1804, 1:1,000), and anti-beta-actin (A2066, 1:1,000) were from Sigma; anti-SAMHD1 (ab67820, 1:1,000), anti-NS3 (ab65407, 1:1,000), and anti-Core (ab2740, 1:1,000) were from Abcam; anti-SREBP-1 (39940, 1:1,000) was from Active Motif; HRP-conjugated goat anti-mouse (ZSGB-Bio, catalog ZB2305, 1:5,000) and goat anti-rabbit (ZSGB-Bio, catalog ZB2301, 1:5,000) were secondary antibodies.

### Gene silencing

siRNAs were transfected into Huh7.5.1 cells seeded into six-well plates by using LipofectamineTM RNAimax (Invitrogen, catalog 13778-150) at the concentration of 50 pmol/well. siRNAs were purchased from JSTBIO; siNT: target sequences 5′-UUC UCC GAA CGU GUC ACG UTT- 3′ and 5′-ACG UGA CAC GUU CGG AGA ATT- 3′; SAMHD1 siRNA: target sequences 5′- CCU CGU CCG AAU CAU UGA UTT -3′ and 5′-AUC AAU GAU UCG GAC GAG GTT -3′.

### qRT-PCR

Virus RNA or cellular RNA was isolated with TRIzol reagents (Invitrogen) according to the manufacturer’s protocols. Quantification of viral RNA was measured by the use of the one-step SYBR PrimeScript RT-PCR kit (Takara). The primer pair (5′-GCGTTAGTATGAGTGTCGTG-3′ and 5′-TCGCAAGCACCCTATCAG-3′) amplifies the 5′ UTR of HCV. The primer pair (5′-ACAATCATGGCAAACGACAA-3′ and 5′-CTTCTCGTTGTGGGCTTCTC-3′) was used to detect JEV RNA. Glyceraldehyde-3-phosphate dehydrogenase (GAPDH) RNA were selected as an internal control to normalize viral RNA through amplifying with primers 5′-ATCATCCCTGCCTCTACTGG-3′ and 5′-GTCAGGTCCACCACTGACAC-3′.

### Progeny virus analysis

After HCVcc infection, part of the culture medium was collected for qRT-PCR or ELISA tests. qRT-PCR analysis was used to quantify copies of HCV RNA extracted from 200 μl of culture medium by RNA extraction kit (Tiandz), and JFH1 mRNA transcribed *in vitro* was set as the standard substance. Some culture medium was directly applied for ELISA (Laibo Bio) to detect HCV core protein released in supernatants. To measure the infection level of progeny virus, 1 ml of culture medium was incubated with naïve Huh7.5.1 cells seeded in six-well plates for another 72 h. Then, cells were harvested for qRT-PCR and immunofluorescence staining to detect HCV RNA and protein symbolizing the infection level of progeny virus.

### Immunofluorescence staining

Cells were firstly fixed in 4% paraformaldehyde and permeabilized with 0.2% Triton X-100 at room temperature. After washing three times with 1× PBS, cells were incubated with primary antibodies for 1 h at room temperature with gentle shaking, then followed by adding Alexa Fluor-conjugated secondary antibodies [donkey anti-mouse antibody (Alexa Fluor 488) and donkey anti-rabbit antibody (Alexa Fluor 555)] for another 1 h after washing. DAPI was used to stain nuclei. Images were recorded with a PerkinElmer Ultra View VoX confocal imaging system.

### Gene chip

The data analysis of gene expression profiling chip is performed by using the Rosetta Resolver System for data preprocessing and Cluster analysis was carried out using Eisen S Laboratory Cluster 3.0 and the TreeView software (http://rana.lbl.gov/EisenSoftware.htm). Principal component analysis (PCA) with ArrayTrack (http://edkb.fda.gov/webstart/arraytrack/), the DAVID 6.7 online database (http://david.abcc.ncifcrf.gov/), and the KEGG pathway database (http://www.genome.jp/kegg/) were used to express change that is greater than twofold that from the genetic analysis of signaling pathways.

### Total cellular cholesterol analysis

Huh7.5.1 cells were seeded in six-well plates and subsequently transfected with or without pSAMHD1. After 24 h, cells were lysed with hypotonic buffer (25 mM Tris-HCl, 5 mM EDTA, 1 mM dithiothreitol, and protease inhibitor cocktail, pH 7.4) and homogenized with a 22-gauge needle. The sample supernatants were collected by centrifugation at 3,000 rpm for 10 min. Total cellular cholesterol was detected by using an Amplex Red Cholesterol Assay Kit (ThermoFisher Scientific) according to the manufacturer’s instructions.

### High content screening

Prior to high content screening (HCS) platform analysis, HCV core protein and SAMHD1 protein in cells were labeled with fluorescence according to immunofluorescence staining. The correlation of fluorescence intensity between SAMHD1 and HCV core protein was detected by the CellInsight CX5 HCS platform (Thermo Fisher Scientific) with a ×10 objective. HCS Studio™ cell analysis software was applied to quantify proteins based on their own signal intensity. A limit was typically set on cells without SAMHD1 overexpressing.

### Construction of the SAMHD1 knock out Huh7.5.1 cell line

SgRNAs targeting SAMHD1 were designed and cloned into the lentiCRISPRv2 backbone. The sgRNA oligos were shown as follows: SAMHD1 sense: caccgCGGAAGGGGTGTTTGAGGGG and antisense: aaacCCCCTCAAACACCCCTTCCGc. The oligos were annealed (5 min at 95°C; 2 min at 85°C; 2 min at 65°C; 2 min at 45°C; 2 min at 25°C) and ligated into lentiCRISPRv2 by the *Bsm*BI restriction site. HEK293T cells were transfected with lentiCRISPRv2-sgRNA targeting SAMHD1, pVSVG, and psPAX2 to produce lentivirus, and then lentivirus containing lentiCRISPRv2-sgRNA targeting SAMHD1 was used to infect Huh7.5.1 cells in the presence of 2 μg/ml puromycin. After 2–4 weeks of monoclonal selection, the level of endogenous SAMHD1 expression was monitored by Western blotting.

### mRNA decay assay

Huh7.5.1 cells seeded in six-well plates were transfected with plasmid expressing SAMHD1 or pcDNA4.0 set as a control for 24 h, and then 5 μg/ml actinomycin D was added into culture medium for the next 12 h. After treatment with actinomycin D, cells were harvested at five consecutive time points (0, 3, 6, 9, and 12 h) for RNA extraction and qRT-PCR analysis. SREBP1 mRNA abundance was detected by a primer pair (5′-GCGCAGATCGCGGAGCCAT-3′ and 5′-CCCTGCCCCACTCCCAGCAT-3′) and normalized to GAPDH mRNA. Curves were fitted to mRNA signal and time by linear regression. mRNA half-lives were calculated using the following equation: t1/2 = ln(2)/k, where k is the elimination rate constant.

### Dual-luciferase reporter gene assay

A predicted regulatory region of a 5′ flanking sequence (−1,500 bp to +55 bp) of SREBP1 was cloned into a pGL3-Basic vector containing a firefly luciferase reporter gene (Promega, Madison, Wisconsin, USA) by the *Kpn* I and *Hind* III restriction sites. The empty pGL3-Basic vector was used as the negative control. The phRL-TK vector containing a Renilla luciferase reporter gene (Promega, Madison, Wisconsin, USA) was used as internal reference in the Dual-luciferase Reporter Assay System. HEK293T cells seeded in six-well plates were co-transfected with 800 ng of SAMHD1 plasmid, 800 ng of the pGL3-Basic-SREBP1 promoter, and 20 ng of phRL-TK by Lipofectamine 2000. pcDNA4.0 was set as a control. After 48 h, cells were lysed and the luciferase activities in cell lysates was monitored by a Dual Luciferase Assay kit (Vazyme, Nanjing, China). The relative luciferase activities are shown as a ratio of the firefly luciferase activity to that of the Renilla luciferase.

### Statistical analysis

All results are shown as the mean ± standard deviation (SD). A two-tailed, unpaired Student’s *t*-test in the GraphPad Prism software was used for statistical analysis. Statistical significance between two groups was marked as follows: *p* < 0.05 (*), *p* < 0.01 (**), and *p* < 0.001 (***). “n.s.” stands for “not significant”.

## Results

### SAMHD1 negatively regulates fatty acid synthesis of the cell

To investigate the role of SAMHD1 in regulating host gene expression, we performed genome-wide gene expression profile analysis of SAMHD1-overexpressing Huh7.5.1 and control cells using microarray gene chip technology, and identified approximately 153 differentially expressed genes (*p* < 0.01, log2 fold change [FC] > 1) (**Supplementary Table S1**). GO pathway enrichment analysis of the differentially expressed genes revealed upregulated mRNAs mainly related to the pathways of cytokine–cytokine interaction, MAPK signaling and chemotactic signaling, and the downregulated mRNAs found in metabolic pathways such as FAs, amino acid metabolism, and terpenoid synthesis.

**Figure 1:**
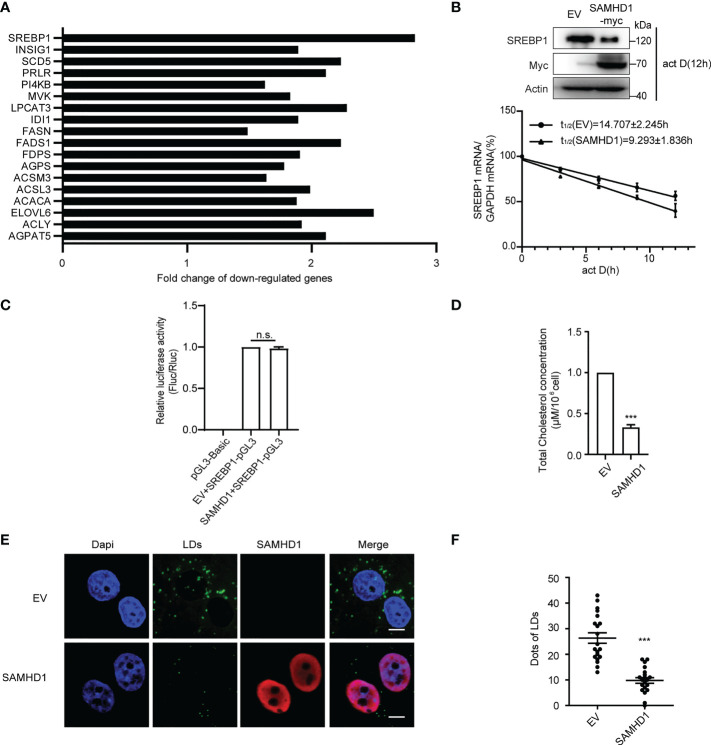
SAMHD1 negatively regulates fatty acid synthesis of cell. **(A)** Plasmid expressing SAMHD1 was transfected into Huh7.5.1 cells for 48 h, pcDNA4.0 was set as a control, and then cells were applied for quantification of genes associated with lipid metabolism by qRT-PCR analysis. The relative changes in gene expression were analyzed by using the 2^−ΔΔCT^ method (Livak method). **(B)** mRNA decay assay was applied to measure the level of SREBP1 mRNA in SAMHD1-overexpressing cells at five consecutive time points (0, 3, 6, 9, and 12 h) in the presence of 5 μg/ml actinomycin D Decay of SREPBP1 mRNA is depicted after normalization to GAPDH mRNA and mRNA half-lives are calculated (mean values ± SD; *n* = 3). The expression of endogenous SREBP1 and extrogenous SAMHD1 proteins at 12 h post-addition of actinomycin D was analyzed by Western blotting. **(C)** HEK392t cells were transfected with plasmid encoding SAMHD1-myc (or pcDNA4.0), pGL3-Basic-SREBP1 promoter, and phRL-TK for 48 h; promoter activities were indicated as a ratio of Firefly luciferase/Renilla luciferase. Data are shown as mean values ± SD (*n* = 3). Values of the EV+SREBP1-pGL3 group arbitrarily set to 1. **(D)** Huh7.5.1 cells were transfected with pSAMHD1 or empty vector for 48 h. Cell lysates were objected to total cholesterol analysis. Data are presented as mean ± SD (*n* = 3). **(E)** SAMHD1 suppresses the formation of LDs. Huh7.5.1 cells with SAMHD1 overexpressing were analyzed by immunofluorescence staining. LDs, SAMHD1, and nucleus were respectively stained with BODIPY493/503 (green), anti-myc antibody (red), and DAPI (blue). Representative images are shown. Bars, 5 μm. **(F)** LD signals were statistical analyzed for 20 randomly selected cells by the use of Image-Pro Plus 7.0C software and plotted as a histogram. Data are shown as mean ± SD. ns, not significant; ***P<0.001.

Since lipid synthesis was reported to participate in host immune and viral replication, we next focus on the investigation of the inhibitory effect of SAMHD1 on expression of genes related to FA metabolism. In agreement with the results of gene expression profile analyses, quantification RT-PCR analysis showed that the expression of SAMHD1 resulted in significant reduction in mRNA abundance of several genes related to the FA bio-metabolic pathway and cholesterol synthesis, including SREBP1, SCD5, and ELOVL6 (**Figure 1A**; **Table S2**). Interestingly, SREBP1 mRNA was the most reduced among them, which encodes a key lipid transcription factor that controls gene expression relevant to FA synthesis. In line with the decreased level of SREBP1 mRNA, the expression of endogenous SREBP1 at the protein level was significantly reduced in the presence of SAMHD1 (**Figure 1B**). To further assess the downregulation of endogenous SREBP1 mRNA by SAMHD1, we applied mRNA decay assay and Dual-luciferase reporter gene assay to measure the impact of SAMHD1 on the stability of mRNA and promoter activity. **Figure 1B** indicates that SREBP1 mRNA had a shorter half-life under the condition of SAMHD1 overexpression, whereas the promoter activity of SREBP1 mRNA was insensitive to exogenous SAMHD1 (**Figure 1C**). This suggests that SAMHD1 reduces the level of SREBP1 mRNA through impairing the stability of mRNA but not mRNA transcription, which contributes to the decrease of SREBP1 protein. Accordingly, the exogenous expression of SAMHD1 significantly downregulates the total level of intracellular cholesterol to approximate 25%–35% of the control group (**Figure 1D**). Furthermore, the immunofluorescence assay revealed that lipid droplets (LDs) were significantly reduced about 50% in the SAMHD1-expressing cells compared with that of control cells (**Figures 1E, F**). These results together suggest an important function of SAMHD1 in negatively regulating cholesterol synthesis and the formation of LDs.

### The antiviral activity of SAMHD1 against flaviviruses

**Figure 2:**
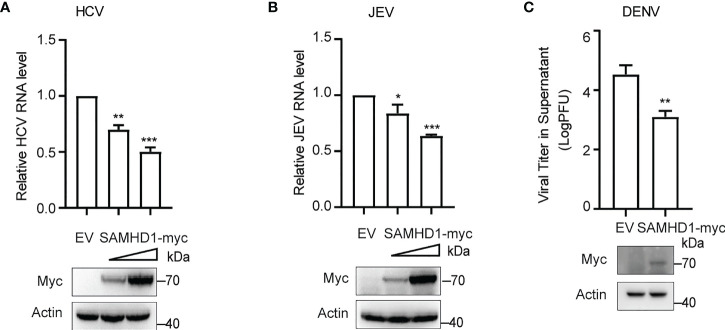
SAMHD1 inhibits the flavivirus infection. Normal Huh7.5.1 cells were transfected with plasmid encoding N-terminal myc-tagged SAMHD1 or pcDNA4.0 for 24 h followed by infection with **(A)** JFH1 HCVcc (MOI = 0.2), **(B)** JEV (MOI = 0.2), and **(C)** DENV (MOI = 0.2), respectively. At 48 hpi, total cellular RNA was isolated by using Trizol reagent for qRT-PCR analysis. HCV and JEV infection levels were evaluated by viral RNA in cells, and viral titer analysis was used for measurement of DENV infection level. Cell lysates were analyzed by Western blotting to determine expression levels of SAMHD1 and β-actin proteins. Data are shown as mean ± SD and depicted as histogram representative of three independent experiments. *P<0.05; **P<0.01, and ***P<0.001.

More evidence indicated the involvement of cellular LDs at different steps of the life cycle of flaviviruses. The inhibitory effect of SAMHD1 on the formation of LDs presented herein inspires us whether SAMHD1 restricts the replication of HCV and other flaviviruses. To address it, we investigated the replication of HCV and several flaviviruses, including JEV and DENV in SAMHD1-expressing cells, which were determined by viral genomic RNA (for HCV and JEV) or virus titer (for DENV). As shown in **Figure 2**, SAMHD1 exhibited different inhibitory effects on the replication of HCV, JEV, and DENV. These results suggest that SAMHD1 may serve as an innate immunity factor to control flavivirus infection. It is worth noting that overexpression of SAMHD1 had no influence on the replication of influenza A virus and enterovirus EV71 (data not shown), suggesting the specificity of its antiviral activity.

### SAMHD1 inhibits HCV replication

**Figure 3:**
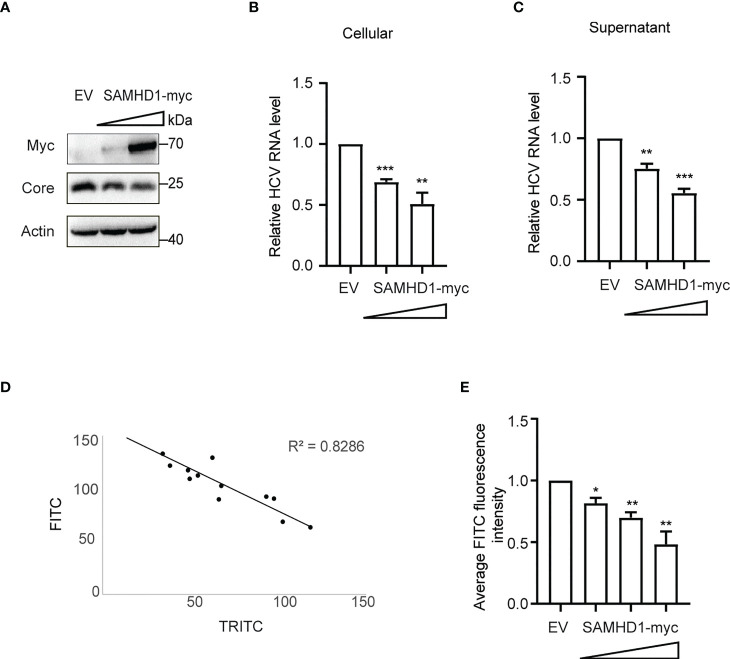
Extrogenous SAMHD1 inhibits HCV replication. Huh7.5.1 cells with extrogenous SAMHD1 overexpression were infected with JFH1 HCVcc (MOI = 0.2) for 48 h. **(A)** Cell lysates were detected by Western blotting to analyze the expression levels of SAMHD1 and HCV core proteins. β-actin was used as a sample loading control. **(B)** Levels of HCV RNA in cells were evaluated by qRT-PCR. **(C)** Levels of infectious progeny viruses in culture supernatants were tested by infecting naïve Huh7.5.1 cells, followed by qRT-PCR analysis of HCV RNA in cells at 72 hpi. All of the data are representative of three independent experiments. For panels **(B, C)** data of SAMHD1 groups are normalized to the control group, whose value is set to 1. **(D)** Huh7.5.1 cells with extrogenous SAMHD1 overexpressing infected with JFH1 HCVcc and processed by immunofluorescence staining. The correlation between fluorescence intensity of SAMHD1 (TRITC) and HCV core proteins (FITC) was detected by high content screening. **(E)** Data of the inhibition on average fluorescence intensity of HCV core protein by different concentrations of extrogenous SAMHD1 are plotted as a histogram. The value of the control group is arbitrarily set to 1 (mean values ± SD; *n* = 3). *P<0.05; **P<0.01, and ***P<0.001.

To further understand the antiviral mechanism of SAMHD1, we investigated the effect of SAMHD1 on viral gene expression and production of HCV. Huh7.5.1 cells transiently expressing N-terminal myc-tagged SAMHD1 were infected with JFH1 HCVcc (MOI = 0.2), followed by quantification of cellular viral protein and RNA level and virus production, using immunoblot and qRT-PCR, respectively. These results showed that overexpressing SAMHD1 exhibited a similar inhibitory effect (approximately 50% inhibition at a higher level of SAMHD1) on viral protein (**Figure 3A**) and RNA (**Figure 3B**) levels and virus production (**Figure 3C**). This suggests that SAMHD1 mainly targets the early step of HCV replication, and impaired virus production most likely is a result of the reduction of viral gene expression. We further examined the anti-HCV activity of SAMHD1 by immunostaining HCV capsid protein in the infected cells. Data of HCS showed significant inhibition of HCV core expression in the SAMHD1-positive cells (**Figure 3D**), and quantification analysis revealed that almost 60% core protein expression was inhibited at the highest SAMHD1 protein level (**Figure 3E**). We also noticed that approximately only 70% of the cells expressed SAMHD1, which shows a significant inhibition of HCV core expression by SAMHD1, and also indicates a probable underestimation of the anti-HCV capacity of SAMHD1 shown by the results in **Figures 3A-C**.

**Figure 4:**
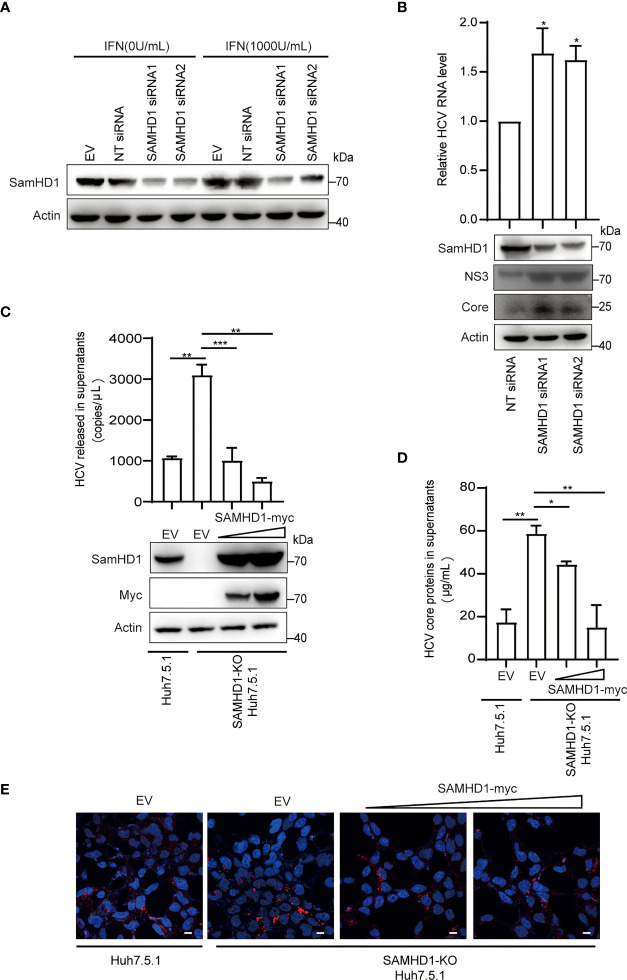
Endogenous SAMHD1 participates in HCV inhibition. **(A)** Huh7.5.1 cells were transfected with 50 pmol/ml siRNAs targeting SAMHD1 or non-targeting siRNA (NT siRNA) for 24 h, and followed by the addition of IFN-α2b (1,000 U/ml) for another 48 h. Cell lysates were analyzed by Western blotting to determine the expression level of endogenous SAMHD1 protein. **(B)** Huh7 cells containing JFH1 subgenomic replicon were transfected with two different siRNAs targeting SAMHD1 for 48 h, and then a part of cells was harvested for measurement of HCV structural or non-structural proteins (core and NS3) by Western blotting. The other part of the cells was applied for total RNA extraction and qRT-PCR analysis. Values are shown as means ± SD (*n* = 3) and the value of the control group is set to 1. **(C–E)** A SAMHD1 knockout Huh7.5.1 cell line constructed by CRISPR-Cas9 technology was used to transfect plasmid expressing SAMHD1 and incubated with JFH1 HCVcc for an additional 48 h; normal Huh7.5.1 cells and pcDNA4.0 were respectively treated as cell control and plasmid control, and HCV RNA and HCV core proteins in culture medium were quantified by qRT-PCR **(C)** and ELISA **(D)**. Data are representative of three independent experiments and shown as a histogram. Expression of endogenous and extrogenous SAMHD1 proteins was verified by Western blotting. Cell culture medium containing progeny virus was incubated with naïve Huh7.5.1 cells for an additional 72 h and detected HCV core proteins by immunofluorescence staining. Representative images are shown (cells > 10), Bars, 5 μm **(E)**. *P<0.05; **P<0.01, and ***P<0.001.

Besides testing the antiviral activity of SAMHD1 overexpression, we also examine the anti-HCV activity of endogenous SAMHD1. SAMHD1 protein expression was strongly induced by IFN-α2b in the Huh7.5.1 cell line and successfully depleted by use of two different small interfering RNAs (siRNAs) (**Figure 4A**). To further confirm the anti-HCV activity of endogenous SAMHD1, we applied these siRNAs targeting SAMHD1 to a Huh7 cell line containing JFH1 subgenomic replicon, followed by the quantification of HCV NS3 and core proteins and RNA level, using immunoblot and qRT-PCR. As **Figure 4B** shows, knockdown of SAMHD1 led to the increase of NS3 and core protein expression and 36% elevation of HCV RNA level, which strongly displayed the inhibition of endogenous SAMHD1. To further confirm the antiviral activity of endogenous SAMHD1, we constructed a SAMHD1 knockout Huh7.5.1 cell line by CRISPR-Cas9 technology and infected with JFH1 HCVcc, followed by detection of progeny virus through qRT-PCR, ELISA, and immunofluorescence analysis. As **Figures 4C-E** show, depletion of endogenous SAMHD1 profoundly rescued HCV RNA proteins released in culture medium by threefold and elevated the infection level of progeny virus. Extrogenous expression of SAMHD1 in SAMHD1-KO Huh7.5.1 cells exhibited a stronger inhibition (approximately 70% inhibition at a higher level of SAMHD1) than in normal Huh7.5.1 cells, which suggests endogenous SAMHD1 indeed possesses antiviral activity. Taken together, both extrogenous and endogenous SAMHD1 are able to negatively regulate the HCV replication.

### The C-terminus of SAMHD1 is required for its anti-HCV activity

**Figure 5:**
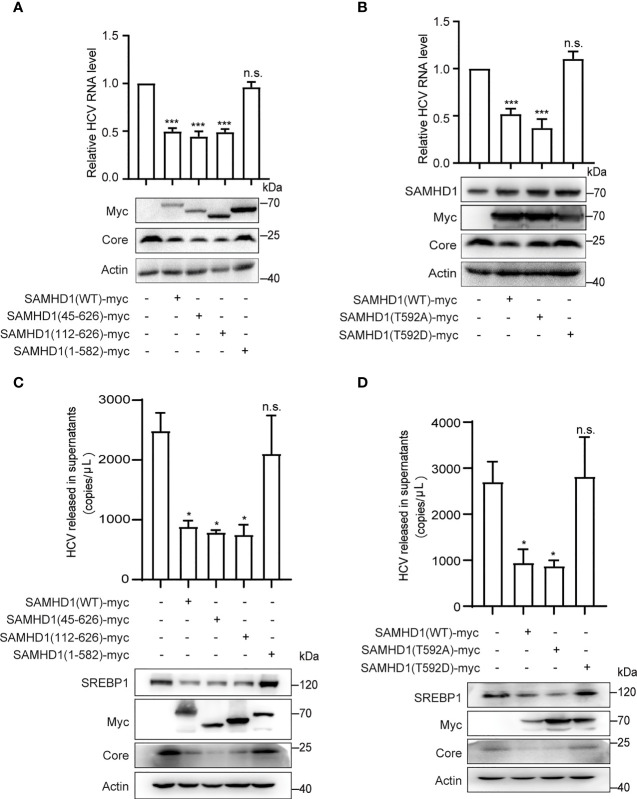
The C-terminus of SAMHD1 is essential for its anti-HCV activity. **(A, B)** Normal Huh7.5.1 cells were respectively transfected with plasmids expressing SAMHD1 truncations [SAMHD1(45–626), SAMHD1(112–626), and SAMHD1(1–582)] **(A)** or SAMHD1 mutants [SAMHD1(T592A) and SAMHD1(T592D)] **(B)**, and followed by infection with JFH1 HCVcc for 48 h. Cell lysates were examined by Western blotting to determine the expression of SAMHD1 and HCV proteins. Replication levels of HCV RNA in infected cells were isolated and quantified by qRT-PCR. **(C, D)** SAMHD1-KO Huh7.5.1 cells were respectively transfected with plasmids expressing SAMHD1 truncations [SAMHD1(45–626), SAMHD1(112–626), and SAMHD1(1–582)] **(C)** and SAMHD1 mutants [SAMHD1(T592A) and SAMHD1(T592D)] **(D)** and infected with JFH1 HCVcc for 48 h. HCV RNA in culture medium was isolated and quantified by qRT-PCR. Cells were harvested for detection of protein expression by Western blotting. All data are representative of three independent experiments and shown as mean ± SD. ns, not significant; *P<0.05, and ***P<0.001.

To determine the domains of SAMHD1 involved in its antiviral activity, we analyzed the inhibitory effect of three SAMHD1 truncations on HCV replication, which consist of residues 45–626, 112–626, and 1–582, representing removal of nuclear localization signal (NLS), SAM, and C-terminal domains, respectively. Huh7.5.1 cells were transfected with plasmid coding for these truncations, followed by HCV infection. Results of Western blotting showed that, similar to wild-type SAMHD1, the expression of 45–626 and 112–626 but not 1–582 truncations significantly reduced the expression of the HCV core protein, which was verified by a twofold reduction in HCV RNA level determined by qRT-PCR (**Figure 5A**), suggesting the importance of SAMHD1 C-terminus in its anti-HCV activity.

It is worth noting that SAMHD1 is regulated by phosphorylation of its C-terminal domain at Thr-592, which annihilates its antiviral function yet has only a small effect on its phosphohydrolase activity. In order to study the relationship between the phosphorylation of Thr-592 and its antiviral activity, we mutated 592 T to A (not phosphorylated) or D (phosphomimetic), and transfected them into Huh7.5.1 cells using SAMHD1 as a positive control followed by infection with JFH1 HCVcc. Interestingly, we found that when we mutated the threonine at position 592 of SAMHD1 to alanine (T→A), which could not be phosphorylated, the expression of core protein was further reduced compared to the wild-type SAMHD1 group (**Figure 5B**). The antiviral effect of SAMHD1 disappeared after we permanently phosphorylated the threonine at position 592 of SAMHD1 to alanine (T→D), indicating that T592 phosphorylation significantly reduced the antiviral effect of SAMHD1.

To rule out the impact of endogenous SAMHD1 and accentuate the antiviral activities of SAMHD1 truncations and mutants, we transfected plasmids expressing SAMHD1 truncations and mutants in SAMHD1-KO Huh7.5.1 cells followed by infection with JFH1 HCVcc. Consistent with the anti-HCV activities in normal Huh7.5.1 cells, only 1–582 truncation and T592D mutant totally lost their inhibition on HCV replication; in addition, they also exerted little effect on SREBP1 protein expression, suggesting a strong correlation between the downregulation of SREBP1 and the inhibition of HCV replication by SAMHD1 (**Figures 5C, D**).

To reinforce such correlation, we also explored functions of SAMHD1 truncations in the formation of LDs by immunofluorescence analysis. Compared with wild-type SAMHD1, 45–626 and 112–626 truncations that are able to inhibit HCV replication reduced the amounts of LDs, whereas the 1–582 truncation weakly obstructed LD formation (**Figure S1**). These data together provide robust evidence on the role of SAMHD1 in SREBP1 downregulation and LD decrease, which are correlated with their antiviral functions.

#### SAMHD1 impairs RNA replication of HCV

**Figure 6:**
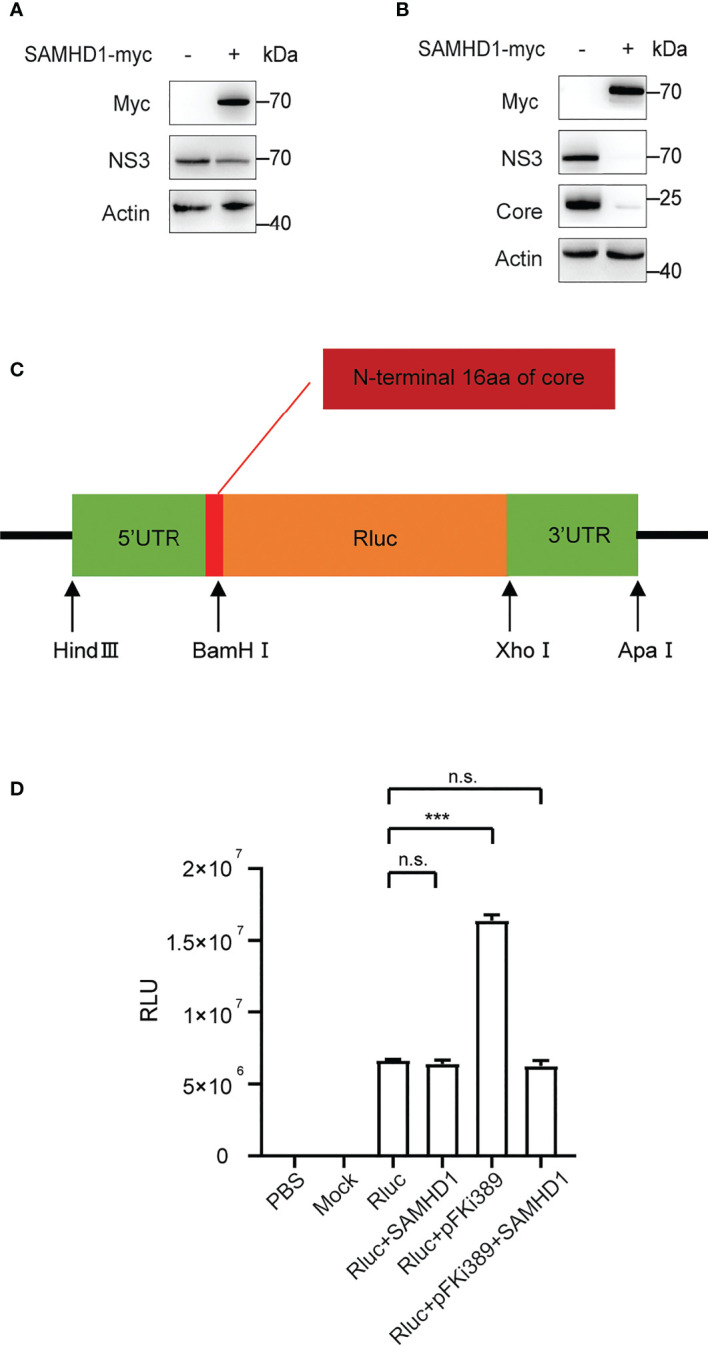
Effect of SAMHD1 on HCV replication and translation. **(A)** Huh7 replicon cells were transfected with plasmid expressing SAMHD1 for 48 h. Cell lysates were analyzed by Western blotting to determine the expression level of NS3 protein. Data are representative of three independent experiments. **(B)** Huh7.5.1 cells were co-transfected with plasmid of SAMHD1 and JFH1 HCVcc RNA transcripted *in vitro*. At 48 h post-transfection, HCV NS3 and core proteins in cells were detected by Western blotting to determine inhibition on HCV replication by SAMHD1. This experiment has been repeated three times. **(C)** Schematic presentation of HCV IRES-mediated luciferase reporter system. **(D)** pFKi389 containing all non-structural proteins was co-transfected with or without pSAMHD1-myc into Huh7.5.1 cells; after 24 h incubation, all cells of test groups but not control groups were transfected with HCV IRES-mediated luciferase mRNA for another 24 h. The values of Renilla luciferase (RLU) activity were measured and used to assess translation or replication level of HCV RNA under circumstances of SAMHD1 overexpression. Data are indicated as mean ± SD (*n* = 3). ns, not significant; ***P<0.001.

Furthermore, we found that overexpressing SAMHD1 in the Huh7 replicon cell line also resulted in inhibition on HCV NS3 expression (**Figure 6A**). This provides evidence supporting that SAMHD1 mainly affects viral gene expression at the stage of transcription or (and) translation. In agreement with the hypothesis, we observed a similar inhibition of viral NS3 and core expression by SAMHD1 in cells transfected with *in vitro* transcripted HCV genomic RNA (**Figure 6B**), which warrants no entry step-involved viral gene expression.

To validate the above hypothesis, we cloned the HCV 5’UTR and 3’UTR into the upstream and downstream of the Renilla luciferase reporter gene, respectively, to construct a reporter gene translation system mediated by HCV IRES (**Figure 6C**). The system contains the complete HCV 5’UTR and 3’UTR and mimics the HCV replication process by providing HCV nonstructural proteins in trans. We obtained the mRNA of Renilla luciferase containing HCV IRES by *in vitro* transcription and then transfect ion into Huh7.5.1 cells with SAMHD1 or pFKi389 (a replicon that contains all non-structural proteins from NS3 to NS5B), or co- transfection of SAMHD1 and pFKi389, and investigated the mRNA levels by testing the luciferase activity (**Figure 6D**). The results showed that the RLU reading of the Rluc+pFKi389 group was doubled compared with the Rluc group, indicating that the luciferase reporter system could be transactivated to mimic HCV RNA replication. First, we found that the expression of SAMHD1 had no significant effect on RLU readings in the absence of HCV nonstructural proteins, suggesting that SAMHD1 does not affect the IRES-mediated translation. However, SAMHD1 drastically reduced the RLU reading when the luciferase reporter system is transactivated. The above results further confirm that SAMHD1 does not affect the IRES-mediated translation process but inhibits the RNA replication process, thereby inhibiting the expression of viral proteins.

### Supplementary lipid counteracts the anti-HCV activity of SAMHD1

**Figure 7:**
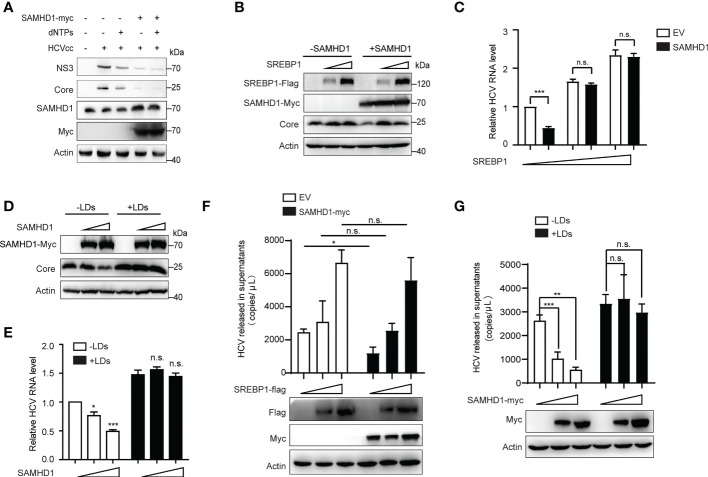
Supplementary lipid rescues HCV replication inhibited by SAMHD1. **(A)** pSAMHD1-myc or pcDNA4.0 was transfected into Huh7 replicon cells with or without the addition of 10 μM dNTPs and cultured for 48 h. Expression levels of HCV core and NS3 proteins were determined by Western blotting (*n* = 3). **(B)** Plasmids encoding SREBP1-flag were co-transfected with pSAMHD1-myc into Huh7.5.1 cells and followed by infection with JFH1 HCVcc (MOI = 0.2) for an additional 72 h. Expression of HCV core protein detected by Western blotting was used to evaluate the HCV replication level. **(C)** HCV RNA in cells was isolated for qRT-PCR analysis. Cells in the control group were transfected with the same amount of empty vector. Values of qRT-PCR are shown as means ± SD (*n* = 3) and the value of the control group is set to 1. **(D)** Huh7.5.1 cells were transfected with pSAMHD1-myc followed by infection with JFH1 HCVcc (MOI = 0.2) in the presence of 100 μg/ml LDs for an additional 72 h. Expression of HCV core protein in cells detected by Western blotting was used to measure HCV infection level. **(E)** HCV RNA in cells was extracted for qRT-PCR analysis. Data are representative of three independent experiments and shown as means ± SD. **(F)** Plasmids encoding SREBP1-flag were transfected into SAMHD1-KO Huh7.5.1 cells with or without extrogenous SAMHD1 expression and followed by infection with JFH1 HCVcc (MOI = 0.2) for an additional 72 h. HCV RNA in culture medium was isolated for qRT-PCR analysis. Values of qRT-PCR are shown as means ± SD (*n* = 3). Expression of extrogenous proteins was detected by Western blotting. **(G)** SAMHD1-KO Huh7.5.1 cells were transfected with pSAMHD1-myc followed by infection with JFH1 HCVcc (MOI = 0.2) in the presence of 100 μg/ml LDs for an additional 72 h. Progeny virus RNA in supernatants was extracted for qRT-PCR analysis. Data are representative of three independent experiments and shown as means ± SD. Expression of extrogenous proteins was analyzed by Western blotting. ns, not significant; *P<0.05, **P<0.01, and ***P<0.001.

To explore if the anti-HCV effect of SAMHD1 is related to its ability to decrease intracellular dNTPs, we examined whether recruitment of dNTPs would rescue HCV replication in SAMHD1-transfected Huh7 replicon cells. Results showed that expression levels of HCV proteins did not increase when compared with that of the control group (**Figure 7A**), suggesting that the antagonism of SAMHD1 on HCV infection is not achieved by reducing intracellular dNTP levels, and other mechanisms may exist.

It was previously reported in the literature that HCV infection can activate the expression of SREBPs, FA synthetase (FASN), and other genes involved in the lipid synthesis and transportation. Inhibition of the activity of SREBPs and FASN blocks the replication of HCV RNA and the production of infectious virus particles. SAMHD1 inhibits the expression of SREBP1, which probably results in inhibiting HCV RNA replication. Based on the above conjecture, we co-transfected the SAMHD1 and SREBP1 plasmids in Huh7.5.1 cells or SAMHD1-KO Huh7.5.1 cells followed by infection with JFH1 HCVcc to observe whether the inhibitory effect of SAMHD1 on HCV changed. As expected, the results of our Western blotting and qRT-PCR assays further confirmed that the supplementary SREBP1 plasmid counteracts the anti-HCV activity of SAMHD1 (**Figures 7B, C**). A similar result was obtained when rescuing LD s (**Figures 7D, E**). Without interference of endogenous SAMHD1, the level of HCV production in the presence of extrogenous SAMHD1 proteins rescued by extrogenous expression of SREBP1 or addition of LDs was more obvious ly observed (**Figures 7F, G**), which suggests that SAMHD1 suppresses the host cholesterol and FA biosynthesis pathways by downregulating the expression of SREBP1 to inhibit HCV replication.

## Discussion

Innate immunity is at the forefront of cellular defense that monitors and recognizes viruses and is characterized by IFN stimulation. SAMHD1 is discovered early as an antiviral ISG, widely existing in eukaryotes and prokaryotes, and highly homologous. At present, the study of the antiviral function of SAMHD1 mainly focuses on retrovirus and some DNA virus, and few studies focus on other kinds of virus (24, 25). Our work expands the antiviral spectrum of SAMHD1 to RNA virus including HCV, JEV, and DENV, suggesting that SAMHD1 has evolved to negatively regulate a wide range of different pathogenic viruses, thus playing a crucial function in innate immunity. We note that SAMHD1 moderately inhibits HCV or JEV compared with HIV or other DNA viruses. One possibility is that these viruses evolved a partial resistance to SAMHD1 during a long evolutionary process (26). The other possibility is that some other host factors induced by type1 IFN, such as CypA, assist in the formation of HCV replicase complex or guard HIV nucleic acids from cytosolic sensors, which counters the inhibition of SAMHD1 (27).

SAMHD1 significantly enhances the antiviral immune response and regulates the IFN-induced inflammatory response involved in the host–virus defense system (28–30). At present, SAMHD1 is considered to restrict HIV-1 reverse transcription by hydrolyzing the majority of cellular dNTPs below the concentration needed for efficient catalysis by viral enzymes through its dNTPase activity. The addition of exogenous dNTPs or knockdown of SAMHD1 partially reverses such inhibition (31). Moreover, HIV Vpx also accelerates ubiquitination of SAMHD1 and is marked for proteasomal degradation (6, 32). However, this strategy of protecting host from virus infection through depletion of dNTPs hardly explains the entirety of SAMHD1’s antiviral functionality (33). Our work suggests a new antiviral strategy of SAMHD1 through impairing lipid synthesis independent of decreasing dNTP pools, providing new evidence supporting the multi-antiviral activities of SAMHD1.

The phosphorylation of SAMHD1 at T592 has been shown to be involved in multiple cellular processes, including tetramer association, cell cycle phase and dissociation, expedited regulatory nucleotide release, and antiviral activity. All types of IFNs share activation of SAMHD1 *via* dephosphorylation at T592 (14, 34, 35). Similarly, our work showed that T592 phosphorylation significantly reduced the anti-HCV effect of SAMHD1. These results together suggest the crucial role of this key site in IFN-mediated innate immunity against different viruses.

IFNs are multifunctional cytokines that widely manipulate intracellular processes to defend virus infection. There is no doubt that viruses may participate in manipulating the cholesterol and FA synthesis to support their replication (36). Viral replication is a high- energy-demanding process; thus, they modulate not only FAs but also cholesterol to provide ATP and use lipids for their nucleic acid production (36–38). Recent studies have shown that an antiviral pathway of IFN seems to be related to impairment of lipid biosynthesis (39). Studies subsequently indicate that IFN can directly inhibit transcription and expression of SREBPs *via* IFNAR1 or stimulate a series of ISGs to realize the inhibition of lipid synthesis (40). We surprisingly discover that SAMHD1 possesses such function of downregulation of lipid production likely through restraining SREBP1 transcription. The inhibition of whole lipid metabolism contributes to the reduction of LDs. Such a cascade of reactions caused by SAMHD1 finally leads to the crush of HCV RNA replication. This discovery has been regarded as a participant in innate immunity and extensively addressed the biochemical functions of SAMHD1. Collectively, SAMHD1 impairing lipid metabolism for viral inhibition is considered as a fresh supplement for cellular innate immunity regulated by IFN.

## Data availability statement

The original contributions presented in the study are included in the article/**Supplementary Material**. Further inquiries can be directed to the corresponding authors.

## Author contributions

SC, LY, and DY conceptualized and supervised the study. QL, XL, FG, and CL performed formal analysis and carried out the investigation. NA, QG, and HS performed the main experiments. SC and DY wrote the original draft and reviewed the manuscript. All authors contributed to the article and approved the submitted version.

## Funding

This work was supported by the National Natural Science Foundation of China 81902075 (to DY) and CAMS Innovation Fund for Medical Sciences 2021-I2M-1-038 (to SC), 2021-I2M-1-030 (to QL), and 2021-I2M-1-043 (to XL).

## Conflict of interest

The authors declare that the research was conducted in the absence of any commercial or financial relationships that could be construed as a potential conflict of interest.

## Publisher’s note

All claims expressed in this article are solely those of the authors and do not necessarily represent those of their affiliated organizations, or those of the publisher, the editors and the reviewers. Any product that may be evaluated in this article, or claim that may be made by its manufacturer, is not guaranteed or endorsed by the publisher.

## Supplementary material

Supplementary Figure 1 Effects of SAMHD1 truncations on the formation of LDs. Huh7.5.1 cells with extrogenous SAMHD1 truncations [SAMHD1(45–626), SAMHD1(112–626), and SAMHD1(1–582)] overexpressing were measured by immunofluorescence staining. LDs, SAMHD1, and nucleus were respectively stained with BODIPY493/503 (green), anti-myc antibody (red), and DAPI (blue). Representative images are shown. Bars, 5 μm.

Supplementary Table 1 Genome-wide gene expression profile analysis of SAMHD1 overexpressing Huh7.5.1 cells

Supplementary Table 2 Description of genes associated with lipid metabolism in **Figure 1A**.
