# Supplementary figures and images for: Interferon-inducible SAMHD1 restricts viral replication through downregulation of lipid synthesis
Source: Front Immunol. 2022 Nov 30;13:1007718. doi: 10.3389/fimmu.2022.1007718 (PMC9755837; doi:10.3389/fimmu.2022.1007718)

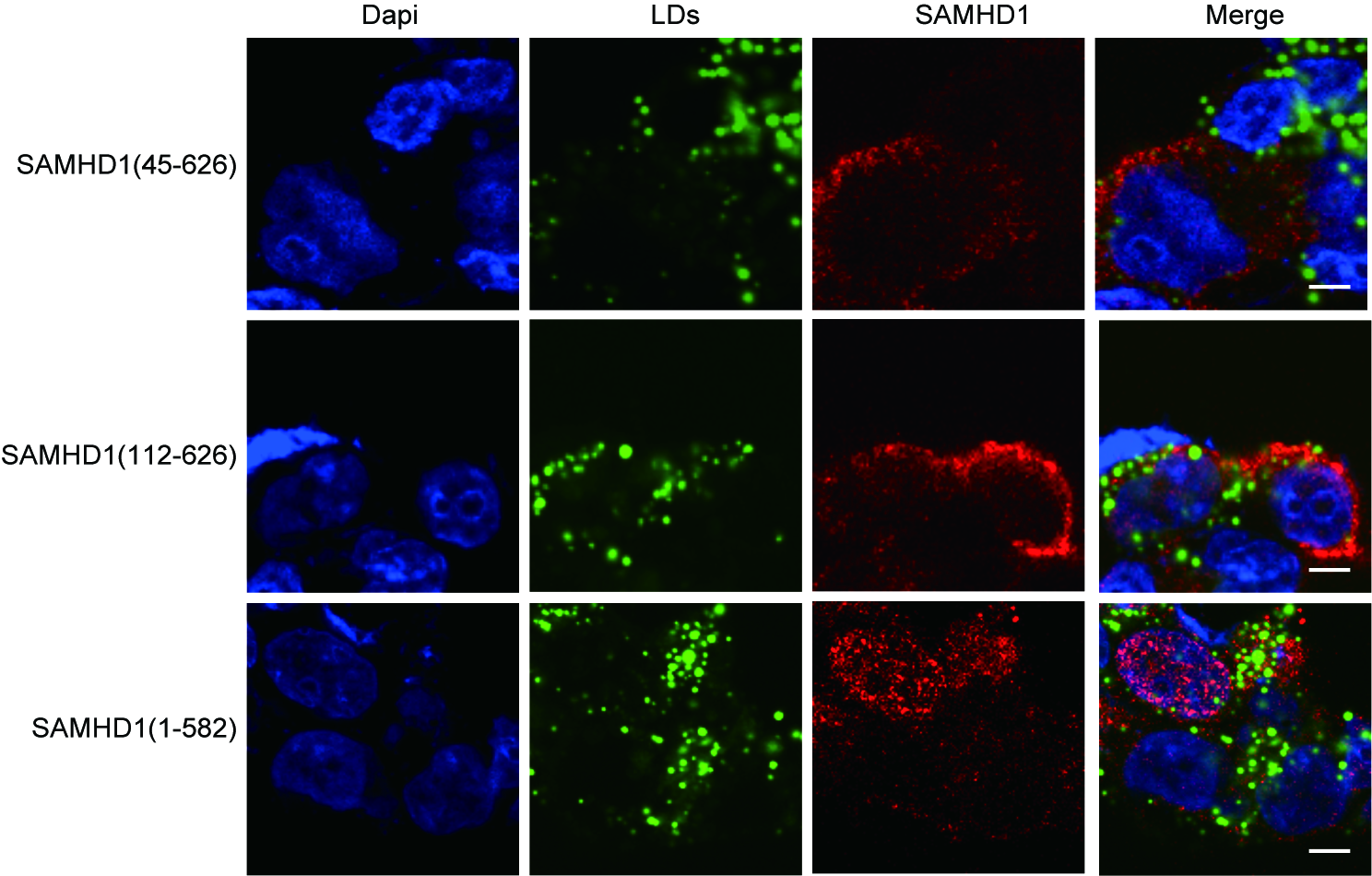

Supplement: Supplementary Figure 1 — Effects of SAMHD1 truncations on the formation of LDs. Huh7.5.1 cells with extrogenous SAMHD1 truncations [SAMHD1(45–626), SAMHD1(112–626), and SAMHD1(1–582)] overexpressing were measured by immunofluorescence staining. LDs, SAMHD1, and nucleus were respectively stained with BODIPY493/503 (green), anti-myc antibody (red), and DAPI (blue). Representative images are shown. Bars, 5 μm. [file Image_1.tif]
